# Supplementary material for: The synaptic ribbon is critical for sound encoding at high rates and with temporal precision
Source: eLife. 2018 Jan 12;7:e29275. doi: 10.7554/eLife.29275 (PMC5794258; doi:10.7554/eLife.29275)
Supplement: Source code 2. [file elife-29275-code2.zip › Live Calcium Imaging analysis/Manual.docx]

1. Load Data (the folder containing andor acquisition) LGS()
2. Reprefix (“1”,”2”) in order to shorten the files name, then keep CD_01 in root and put CD_02 in folder 2
3. Windows- procedure windows- autobatchprocess.ipf and execute this code


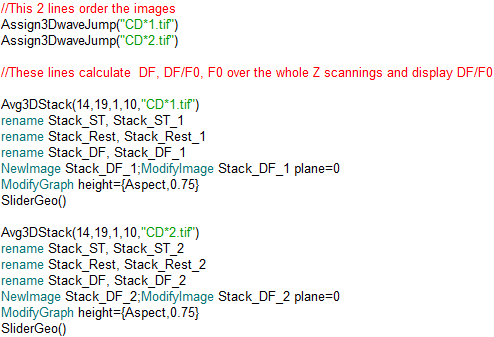


It resorts data sequence by the step-up order on Z( Assign3DWaveJump(Key) ) , assign data to folders, and calculates ΔF,ΔF/F­­_0_, F_0_ over the whole Z scannings ( Avg3DStack(initial.stim.time**,** end.stim.time**,** initial.rest.time**,** end.rest.time**,** key words in desired waves) )

It will create 6 stacks (3 per stimulus)

1. Stack_ST_1 and 2
2. Stack_Rest_1 and 2
3. Stack_DF_1 and 2

And 2 graphs in Packages folder : graph 1 and 2

1. load patchmaster file by PPT into “PatchClamp” (data browser, load experiment)
2. In each folder, the patch clamp data should be once more assigned to the folders of different optical sections
   ( AssignPatchData(i) , I is the series number of patch clamp data, normally 2 )

( This procedure includes series resistance correction ( RSCAcrossFolders(frame interval)) )

It will create in each FV section: VmonCor_1 and 2 and VmonSlow_1 and 2

1. Open waves “Stack_DF1“, “Stack_DF2“
2. Do Slidergeo()
3. Menu “Hot Spots”-> “Square ROI auto processing”

First, find the hotspots in the Stack_DF1 and 2 files.

1. On Stack_DF1, Move cursor A to maximum intensity position (x y and z) of the spot, press the button “Square ROI auto processing” (average of **9 px**)

It creates in the desired stack: spot n and square x_n

- 1. It automatically generate a green annotation, move this annotation nearby the spot

1. Find the same spot and do the same thing on “Stack_DF2” it creates in the desired stack: spot n+1 and square x_n+1 (**same stack than DF1**)
   ** MUST DO IT AS THE ORDER DF1->DF2DF1->DF2->…… Keep the annotation numbers in DF1 odd and those numbers in DF2 even.*
2. Repeat 4), 5) and 6) for all the ribbons and hotspots. At the end, it will look something like that


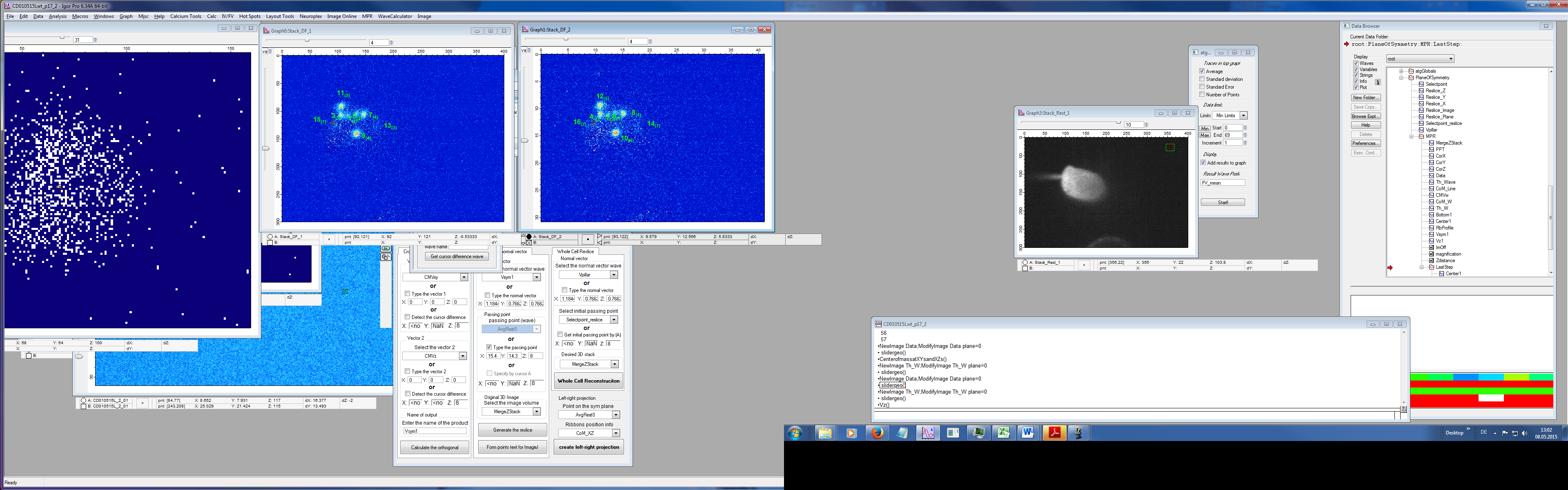


1. After all the spots are specified, do FindSpotsROI(). This command automatically finds all the spots, calculate their FV traces from images, and assign them into folders. It creates:

Spot_X: copy/past spot n and square x_n, spot n+1 and square x_n+1, CD_2_X_1 and 2.tif, VmonSlow_1 and 2

| **(!)** Check the Rs for each plane:  - Localize the ephys info of each plane containing hotspots (in the Patchclamp:2 folder)  - Duplicate each “Section_x” folder and copy them to the folder “out”  - Put the arrow in the folder out and run CheckRs(). Check whether it is adjusted to your threshold. If not, look for the individual section to see which Spot you have to discard. If it is higher, than your criteria, this cell cannot be used. |
| --- |

1. Move the folder for hotspots (Spot_1….) to the FV
2. Open wave “Stack_Rest_1”, choose a super dark corner as the background, and use the ROI tool in menu “Image” to specify this super dark region to be the background ROI. Remember to change the wave name “M_ROIMask” to “BGROI”. Also move the BGROI to folder “FV”
3. Do AssembleAllFV() to assemble the 2 FV traces of a spot.

IMPORTANT: Before running the following function, check if the range for Fmax is correct. It should match the peak of the increase in fluorescence.

It creates in each spot folder:

Copy/past BGROI

Creates Avg_square x_n and n+1 (Fluo against the voltage)

Ratio_square x_n and n+1 (The same but baseline 0)

Avg_Spot 0 (Fluo against the time)

Vslow_spot 0 (Voltage cmd against time)

Ratio_Spot 0 (Fluo against time averaged with BGROI)

1. Do FVinfoOutput(n). This macro automatically refines the zigzag raw FV traces, generate the initial guess for fitting the sigmoidal equation, and then fit it.
   1. There are 2 different ways (procedures) to generate initial guess. In some waves the fitting doesn’t work properly, try the other initial-guessing generation might make the fitting work. To select each, change n: 1: Standard method with a fix Vr value, 2:New method.

The preferred one is number 1. You can change the Vr value according to your experimental data in RefineFV_fixvr(). I first run the FVinfoOutput(1) with the standard value calculated by TL in a series of recordings in optimal conditions, 65mV. Then, I run SigmOut(), which will put in a folder the FV traces and the sigmoidal fitting. I plot all the FV traces together and I average them (Macros 🡪 Average trace from graph). I plot the average against voltage and then calculate the Vr of this averaged trace (Cursor A and B in the range around 20-40mV 🡪 Right click 🡪 Quick fit 🡪 line; then a and b (linear function: y=a+bx) will appear.Calculate the VFr Vr=a/b; so print a/b ). Run again FVinfooutput(1) with this modified value (Modify it in RefineFV_fixvr()).

The Cainfo_Cor wave contains info of each spot:

1^st^ column: DFmax/Fo (wavemax(DF/Fo))

2^nd^ column: Fractional Activation 0

3^rd^ column: Fractional Activaction max

4^th^ column: Vh

5^th^ column: k
