# Supplementary figures and images for: The synaptic ribbon is critical for sound encoding at high rates and with temporal precision
Source: eLife. 2018 Jan 12;7:e29275. doi: 10.7554/eLife.29275 (PMC5794258; doi:10.7554/eLife.29275)

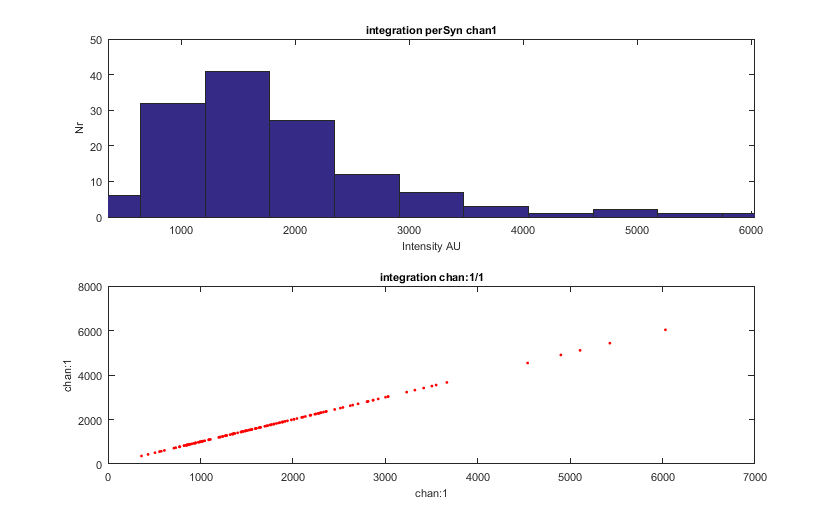

Supplement: Source Code 4. [file elife-29275-code4.zip › Count and intensity of immunofluorescent spots/Example Spot Intensity-1-1-Intensity.TIF]

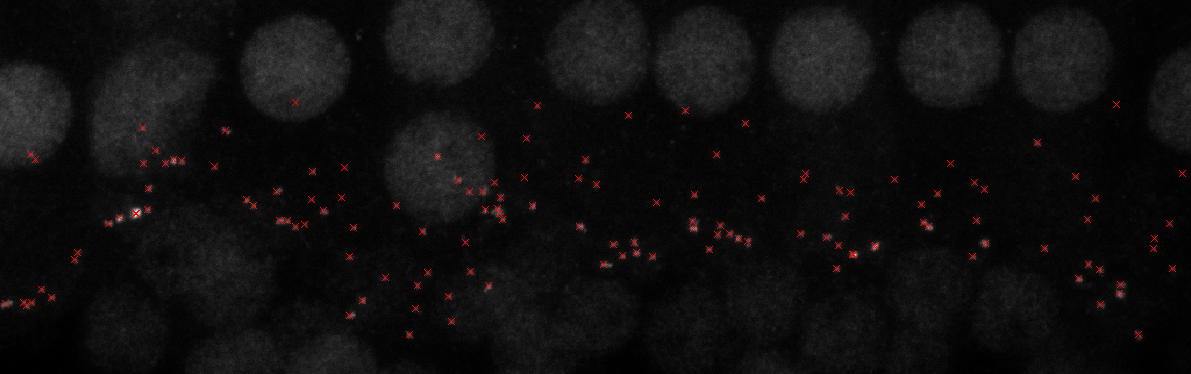

Supplement: Source Code 4. [file elife-29275-code4.zip › Count and intensity of immunofluorescent spots/Example Spot Intensity-1-IntensROIXYpic.TIF]

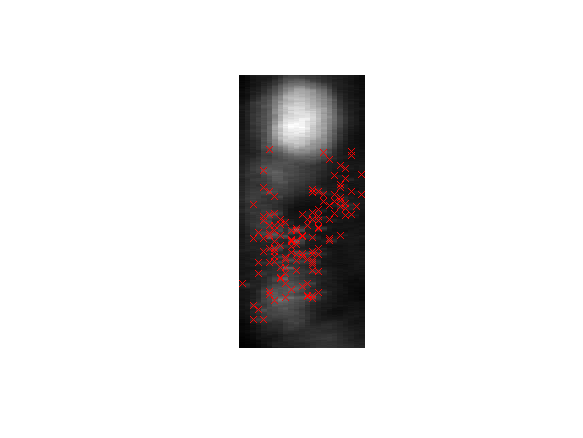

Supplement: Source Code 4. [file elife-29275-code4.zip › Count and intensity of immunofluorescent spots/Example Spot Intensity-1-IntensROIYZpic.TIF]

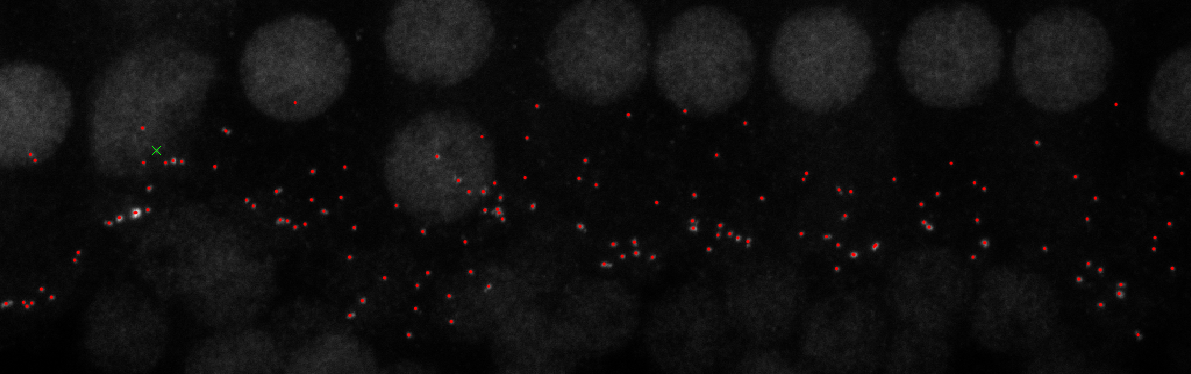

Supplement: Source Code 4. [file elife-29275-code4.zip › Count and intensity of immunofluorescent spots/Example Spot IntensityMbHand Spots1.TIF]
