# Supplementary material for: The synaptic ribbon is critical for sound encoding at high rates and with temporal precision
Source: eLife. 2018 Jan 12;7:e29275. doi: 10.7554/eLife.29275 (PMC5794258; doi:10.7554/eLife.29275)
Supplement: Supplementary file 1. — The table depicts the modified tomogram analysis performed according to Jung et al., 2015a displaying the mean ± (S.E.M.), (S.D.), p-values, sample size and statistical tests used to compare RBEWT/WT and RBEKO/KO. Refer to Figure 3—figure supplement 1 for the graphs. [file elife-29275-supp1.docx]

**Supplementary file 1**

|  | **Parameters** | **RBE^WT/WT^**  *(n* = 9 ribbons*,*  N = 3 animals)  **[mean ± (S.E.M.), (S.D.)]** | **RBE^KO/KO^**  *(n* = 11 PDs*,*  N = 3 animals)  **[mean ± (S.E.M.), (S.D.)]** | ***p-values*** | **Statistical tests** |
| --- | --- | --- | --- | --- | --- |
| **Membrane-proximal SVs**  **(MP-SVs)** | SV number | 12.33 ± (0.65), (1.93) | 7.18 ± (0.81), (2.71) | ***0.0001*** | Mann-Whitney-Wilcoxon test |
|  | Fraction of tethered SVs | 0.61 ± (0.04), (0.12) | 0.74 ± (0.05), (0.17) | *0.06* | t-test |
|  | Fraction of SVs with single tether | 0.58 ± (0.03), (0.10) | 0.51 ± (0.60), (0.19) | *0.64* | Tukey’s test |
|  | Fraction of SVs with multiple tethers | 0.03 ± (0.01), (0.04) | 0.22 ± (0.03), (0.11) | ***0.008*** |  |
|  | ***p-values*** | ***4.87E-10*** | *4.21E-05* |  |  |
|  | Diameter of SVs | 48.20 ± (0.51), (1.52) | 50.05 ± (0.80), (2.57) | *0.06* | t-test |
| **Ribbon/PD-associated SVs (RA/PDA-SVs)** | SV number | 26.33 ± (2.30), (8.71) | 8.45 ± (1.20), (3.98) | ***3.005E-05*** | Mann-Whitney-Wilcoxon test |
|  | Fraction of tethered SVs | 0.70 ± (0.06), (0.18) | 0.80 ± (0.05), (0.18) | *0.10* | t-test |
|  | Diameter of SVs | 50.07 ± (1.00), (2.93) | 49.82 ± (0.80), (2.67) | *0.68* | Mann-Whitney-Wilcoxon test |

**Supplementary file 1: Modified tomogram analysis**

The table depicts the modified tomogram analysis performed according to Jung et al. (2015a) displaying the mean ± (S.E.M.), (S.D.), *p*-values, sample size and statistical tests used to compare RBE^WT/WT^ and RBE^KO/KO^. Refer to Figure 3-figure supplement 1 for the graphs.

**Supplementary file 2**

|  |  | **R_s_** | | **R_m_** | | **I_leak_** | | **C_slow_** | |
| --- | --- | --- | --- | --- | --- | --- | --- | --- | --- |
|  |  | RBE^WT/WT^ | RBE^KO/KO^ | RBE^WT/WT^ | RBE^KO/KO^ | RBE^WT/WT^ | RBE^KO/KO^ | RBE^WT/WT^ | RBE^KO/KO^ |
| **Figure 5** | **ruptured (A,B,C)** | 11.34 ± 0.89 MΩ | 10.47 ± 0.87 MΩ | 437 ± 55 MΩ | 535 ± 168 MΩ | -21.19 ± 2.50 pA | -19.43 ± 1.73 pA | 11.81 ± 0.28 pF | 12.53 ± 0.28 pF |
|  | **perforated (D)** | 29.33 ± 2.04 MΩ | 27.22 ± 1.85 MΩ | 1598 ± 184 MΩ | 1532 ± 79 MΩ | -8 ± 1.04 pA | -7.44 ± 0.74 pA | 12.19 ± 0.53 pF | 12.76 ± 0.27 pF |
| **Figure 6** | **ruptured** | 13.75 ± 0.92 MΩ | 13.96 ± 0.82 MΩ | 400 ± 46 MΩ | 406 ± 79 MΩ | -28 ± 3.73 pA | -26 ± 2.66 pA | 10.63 ± 0.29 pF | 10.88 ± 0.26 pF |
| **Figure 7** | **perforated (A-C)** | 28 ± 2.23 MΩ | 25.11 ± 1.66 MΩ | 1373 ± 44 MΩ | 1510 ± 76 MΩ | -7.67 ± 0.69 pA | -8 ± 0.81 pA | 11.93 ± 0.49 pF | 12.76 ± 0.29 pF |
|  | **perforated (D)** | 23.51 ± 0.91 MΩ | 22.08 ± 0.94 MΩ | 879 ± 51 MΩ | 909 ± 40 MΩ | -16.54 ± 1.08 pA | -16.67 ± 1.56 pA | 9.04 ± 0.17 pF | 9.94 ± 0.20 pF |
|  | **perforated (E-F)** | 19.39 ± 1.58 MΩ | 18.67 ± 1.17 MΩ | 1387 ± 138 MΩ | 1226 ± 76 MΩ | -28.02 ± 2.41 pA | -28.11 ± 2.12 pA | 9.13 ± 0.18 pF | 9.57 ± 0.45 pF |
|  | **perforated (G-H)** | 18.16 ± 1.30 MΩ | 18.61 ± 1.35 MΩ | 1260 ± 121 MΩ | 1031 ± 62 MΩ | -23.80 ± 2.46 pA | -21.54 ± 2.70 pA | 9.18 ± 0.17 pF | 9.32 ± 0.16 pF |

**Supplementary file 2: Passive electrical properties of patch-clamp recording experiments**

The table shows the mean ± (S.E.M.) of the passive electrical properties across all ruptured and perforated patch-clamp recording experiments in RBE^WT/WT^ and RBE^KO/KO^ conditions.
